# Preferred Features of E-Mental Health Programs for Prevention of Major Depression in Male Workers: Results From a Canadian National Survey

**DOI:** 10.2196/jmir.5685

**Published:** 2016-06-06

**Authors:** JianLi Wang, Raymond W Lam, Kendall Ho, Mark Attridge, Bonnie M Lashewicz, Scott B Patten, Alain Marchand, Alice Aiken, Norbert Schmitz, Sarika Gundu, Nitika Rewari, David Hodgins, Andrew Bulloch, Zul Merali

**Affiliations:** ^1^ Department of Psychiatry University of Calgary Calgary, AB Canada; ^2^ Department of Community Health Sciences University of Calgary Calgary, AB Canada; ^3^ Mathison Centre for Mental Health Research & Education Hotchkiss Brain Institute University of Calgary Calgary, AB Canada; ^4^ Department of Psychiatry University of British Columbia Vancouver, BC Canada; ^5^ Department of Emergency Medicine University of British Columbia Vancouver, BC Canada; ^6^ Digital Emergency Medicine University of British Columbia Vancouver, BC Canada; ^7^ Attridge Consulting Inc. Minneapolis, MN United States; ^8^ School of Industrial Relations Public Health Research Institute University of Montreal Montreal, QC Canada; ^9^ School of Rehabilitation Therapy Queens University Kingston, ON Canada; ^10^ Department of Psychiatry McGill University Montreal, QC Canada; ^11^ Workplace Mental Health Program Canadian Mental Health Association - National Toronto, ON Canada; ^12^ Workplace Mental Health Mental Health Commission of Canada Ottawa, ON Canada; ^13^ Department of Psychology University of Calgary Calgary, AB Canada; ^14^ Department of Psychology University of Ottawa Ottawa, ON Canada; ^15^ Institute of Mental Health Research The Royal University of Ottawa Ottawa, ON Canada; ^16^ Department of Psychiatry University of Ottawa Ottawa, ON Canada

**Keywords:** depression, Internet, prevention, e-mental health programs, design features, men

## Abstract

**Background:**

Major depression is a prevalent mental disorder and imposes considerable burden on health and productivity. Men are not immune to major depression, yet they often delay seeking help because of perceived stigma and gender norms. E-mental health programs hold potential for early prevention of major depression. However, we have little knowledge about men’s preferences for design features of e-mental health programs.

**Objectives:**

The objective of this study was to (1) estimate and compare the proportions of Internet use for medical information, preferred design features, and likely use of e-mental health programs; (2) examine factors associated with the likely use of e-mental health programs; and (3) understand potential barriers to the use of e-mental health programs among Canadian working men, who were at high risk of a major depressive episode (MDE).

**Methods:**

A cross-sectional survey in 10 Canadian provinces was conducted between March and December 2015. Random digit dialing method was used through household landlines and cell phones to collect data from 511 working men who were at high risk of having an MDE and 330 working men who were at low risk of having an MDE.

**Results:**

High-risk men were more likely to endorse the importance of accessing health resources on the Internet than low-risk men (83.4% vs 75.0%, respectively; *P*=.01). Of the 17 different features assessed, the top three features most likely to be used by high-risk men were: “information about improving sleep hygiene” (61.3%), “practice and exercise to help reduce symptoms of stress and depression” (59.5%), and “having access to quality information and resources about work stress issues” (57.8%). Compared with men at low risk for MDE, men at high risk for MDE were much more likely to consider using almost every one of the different design features. Differences in preferences for the design features by age among men at high risk of MDE were found only for 3 of 17 features. Differences in preferences for design features between English- and French-speaking participants were found only for 4 out of the 17 features. Analysis of qualitative data revealed that privacy issues, perceived stigma, ease of navigation, personal relevance, and lack of personal interaction, time, and knowledge were identified as barriers to the use of e-mental health programs in working men who were at high risk of MDE.

**Conclusion:**

E-mental health programs may be a promising strategy for prevention of depression in working men. Development of e-mental health programs should consider men’s preferences and perceived barriers to enhance the acceptability of this approach.

## Introduction

Major depression is a prevalent mental disorder. In Canada, the annual prevalence of major depressive disorder was 3.9% based on the DSM-IV criteria [1]; in the United States, the 12-month prevalence was 6.6% [2]. Major depression is disabling and significantly affects workers’ health and productivity. Depression alone accounts for 2.5% of the global burden of disease and is among the largest single causes of disability worldwide (8.2% of all years lived with disability globally) [3]. US workers with depression cost an estimated US $44.01 billion per year in lost productivity [4]. Epidemiological studies have consistently found that women are more likely to have major depression than men. However, men are not immune to depression. Canadian national data showed that annually, 2.8% of adult men have a major depressive disorder [1]. One of the severe consequences of having an MDE is suicide. Canadian and US national data showed that 75%-80% of all suicides were male [5,6].

Given its considerable effect on health, productivity, and lives, there is a pressing need for innovation in prevention of major depression in male workers. However, this is a challenging endeavor. In the workplace, risk factors for having an MDE differ for men and women [7-9]. For instance, job strain, family-to-work conflict, and job insecurity appear to be more prominent risk factors for MDE among men than among women [7,8]. Men are less likely than women to seek help and to disclose depressive symptoms and often delay seeking help until symptoms become severe, which compounds these risks. Men tend to socialize, to be emotionally stoic, and exemplify traditional masculine characteristics such as independence, self-reliance, and dominance [10]. Men are concerned about the perceived negative judgments from family, friends, and coworkers if they access treatment for depression. These gender-specific experiences, together with the limited knowledge of effective interventions, call for innovative solutions tailored for men. One of the burgeoning solutions that has attracted considerable interest is e-mental health.

E-mental health is “the use of information and communication technologies to support and improve mental health, including the use of online resources, social media, and mobile phone apps” [11]. E-mental health self-help services enable users to learn more about their mental health conditions and empower them to strengthen their self-management and improve their health. Most e-mental health treatment programs were developed based on the models of cognitive behavioral therapy (CBT) or interpersonal therapy (IPT) that have been proven to be effective in depression and anxiety [12]. A review by Christensen and Petrie showed that by 2013, 62 Web-based mental health interventions and 11 mobile apps had been developed [13]. Lal and Adair found 91 peer-reviewed publications on the application of e-mental health interventions between 2000 and 2010 [14]. Thus far, randomized controlled trials of e-mental health interventions on depression have included individuals with clinical depression or those above a threshold of a depression rating scale [14]. Consistent with public health mandates to reduce the burden of depression, it is important that e-mental health not only addresses needs of those with depression or those who are above a threshold depression rating, but also advances capacity for secondary prevention [15], by identifying high-risk individuals and intervening to prevent early symptoms from progressing into an MDE. Additionally, existing e-mental health programs have not been designed and evaluated using a gender lens. Given the gender norms, the extent to which men accept e-mental health programs is unknown. Effectiveness of the program and its acceptability to users constitute the foundation for scalable and sustainable program implementation. Therefore, as part of our BroMatters study (www.bromatters.ca), we conducted a cross-sectional survey among working men, some of whom were at high risk of MDE, to understand their preference for design features of e-mental health programs.

The objectives of this analysis were to (1) estimate and compare the proportions of Internet use for medical information, preferred design features, and likely use of e-mental health programs, (2) examine factors associated with the likely use of e-mental health programs, and (3) understand potential barriers to the use of e-mental health programs among working men who were at variable risk levels of MDE.

## Methods

### Study Design and Recruitment

A cross-sectional survey was conducted between March and December 2015. The target population of the survey included Canadian working men who: (1) were aged 18 years or older, (2) did not have an MDE in the past 12 months, (3) were at high risk of an MDE at the time of interview (a low-risk sample was also obtained for comparison), (4) were working at the time of the survey, and (5) have no language barriers to either English or French. Because of the vast geographic area of Canada, participants were recruited using the random digit dialing method by the Bureau of Professional Interviewers (BIP), located in Montreal, Canada. The BIP has access to household telephone numbers across the country and to a validated mobile phone number database, and its interviewers can conduct interviews in both English and French. The Conjoint Health Research Ethics Review Board of University of Calgary approved the study.

Once a household was reached, the household contact was asked to retrieve or provide contact information (eg, a first name) of the household residents who are men and are currently working. If there was more than one potentially eligible individual in the same household, one of them was randomly selected. Once the prospective participant was fully informed about the objectives and procedures of the study, oral consent was obtained to proceed with the interview. Participants were first administered a risk calculator for MDE to estimate their probability of having an MDE in the future. The definition of high risk is described below. The number of high-risk participants in each age group was proportional to the age distribution of Canadian male working population in 2014, provided by Statistics Canada.

From March to December 2015, 49,500 calls were made. A majority of the calls (47,648, 96.2%) were not valid (not in service, fax or modem, answering machine, language barriers, ineligibility, duplications, refusal before eligibility was assessed). Among 1852 eligible participants, 596 (32.1%) refused to participate after eligibility verification; 842 provided complete data (45.4%). The remaining included incomplete interviews or scheduled call-backs not in study period (22.5%). After removing 1 duplication, 841 participants were included in the analysis, including 511 men who were at high risk of having major depression and 330 who were at low risk of having major depression.

### Measurements

A multivariable riskprediction algorithm for major depression in men was administered to estimate the risk (probability) of having an MDE in the next 4 years for each participant [16]. This risk prediction model was designed to be used in individuals who did not have an MDE. Based on the participant’s exposure to a set of key risk factors (predictors) in the model, the algorithm can generate the absolute risk or probability of having an MDE in the next 4 years, analogous to the Framingham riskprediction algorithm for coronary heart disease [17,18]. The risk-prediction algorithm for MDE in men was developed and validated using data of 4737 Canadian men who were aged 18 years or older and did not have an MDE in the past 12 months [16]. The risk-prediction algorithm contains 15 predictors including age, personal and family history of MDE, childhood trauma, ongoing stress and life events, and antidepressant or sleeping pill use in the past month. The predictive power of the risk-prediction algorithm was measured using C statistics (C=0.7953) [16], which is equivalent to the area under the curve when the outcome is binary. The model had excellent calibration with data as indicated by the Hosmer–Lemeshow test and a visual comparison between the predicted and observed risks by decile risk groups [16]. In our study, >6.51% were identified as high risk, which represents the top two decile risk groups in the Canadian male population. Predicted risk lower than 6.51% was defined as low risk. Internet use was assessed using questions from the 2012 Canadian Internet Use Survey conducted by Statistics Canada [19].

Preferred design features of e-mental health program questions were developed by BroMatters team members. Participants were asked questions such as, “We want to hear your opinion about e-mental health programs for dealing with work and stress issues. E-health is defined as …, For the following features, please indicate how likely it is that you would use them *.* ” In all, 17 questions about design features were asked. For each question, participants answered on a 5-point Likert scale ranging from very likely to very unlikely. Open-ended questions were asked about any other features they may want in an e-mental health program, whether the participant and his male coworkers may use an e-mental health program to deal with work stressand what makes it difficult to use an e-mental health program. Administering the questions and instruments to eligible participants took an average of 22 min. Participants who completed the survey received a CAN $20 gift card as a token of appreciation.

### Statistical Analysis

The background characteristics and proportions of likely use of design features were estimated and compared between men who were at high or low risk of having an MDE using the chi-square test. Among men who were at high risk of having an MDE, the percentages were also estimated and compared by age groups and by language used in the interview (English vs French) using a chi-square test. All analyses were conducted using the statistical program STATA 14.0 (StataCorp, College Station, TX, USA). Tests were considered statistically significant when *p* was less than .05. With this level of probability and a sample size of 841, the study had a statistical power of 0.89 to detect a small effect size (Cohen d) of 0.20.

## Results

### Participants’ Characteristics

The demographic and socioeconomic characteristics of the participants are summarized in Table 1. Men who were at high risk of having an MDE had characteristics similar to those at low risk, except that high-risk men were younger and were more likely to have reported work function impairment (*P*<.001).

**Table 1:** Demographic and socioeconomic characteristics of the participants overall and by risk levels of having major depression.

Variable		Total (N=841), n (%)	High risk (n= 511), n (%)	Low risk (n=330), n (%)	*P*
Age categories (years)					<.001
	18-29	102 (12.1)	73 (14.3)	29 (8.8)	
	30-49	444 (52.8)	284 (55.6)	160 (48.5)	
	50-64	254 (30.2)	140 (27.4)	114 (34.5)	
	>65	41 (4.9)	14 (2.7)	27 (8.2)	
Mean age (SD)		44.3 (13.7)	42.0 (12.2)	47.8 (15.0)	
Marital status					.14
	Married or Common-law	647 (77.0)	389 (76.1)	258 (78.4)	
	Divorced or separated/widowed	43 (5.1)	22 (4.3)	21 (4.4)	
	Single	150 (17.9)	100 (19.6)	50 (15.2)	
Personal income ($)					.60
	<30,000	99 (12.2)	60 (12.1)	39 (12.4)	
	30,000 to <60,000	238 (29.4)	152 (30.6)	86 (27.4)	
	60,000 to 80,000	171 (21.1)	98 (19.7)	73 (23.3)	
	>80,000	303 (37.4)	187 (37.6)	116 (36.9)	
Educational levels					.15
	Above high school	65 (7.7)	43 (8.4)	22 (6.7)	
	High school	166 (19.7)	107 (20.9)	59 (17.9)	
	College	265 (31.5)	167 (32.7)	98 (29.7)	
	University or higher	345 (41.0)	194 (38.0)	151 (45.8)	
Employment					.70
	Employee	673 (80.6)	413 (81.5)	260 (79.3)	
	Self-employed	159 (19.0)	92 (18.2)	67 (20.4)	
	Family business no pay	3 (0.4)	2 (0.4)	1 (0.3)	
Job type					.16
	Full time	692 (82.3)	434 (84.9)	258 (78.2)	
	Part time	71 (8.4)	37 (7.2)	34 (10.3)	
	Seasonal	37 (4.4)	18 (3.5)	19 (5.8)	
	Contract	36 (4.3)	19 (3.7)	17 (5.2)	
	Other	5 (0.6)	3 (0.6)	2 (0.6)	
Size of company or work site					.78
	<50	445 (53.6)	276 (54.2)	169 (52.5)	
	50-499	243 (29.2)	149 (29.3)	94 (29.2)	
	>500	143 (17.2)	84 (16.5)	59 (18.3)	
Provinces					.22
	British Columbia	47 (6.3)	33 (7.1)	14 (5.0)	
	Alberta	70 (9.4)	52 (11.2)	18 (6.4)	
	Saskatchewan	30 (4.0)	18 (3.9)	12 (4.3)	
	Manitoba	31 (4.2)	20 (4.3)	11 (3.9)	
	Ontario	293 (39.3)	170 (36.6)	123 (43.4)	
	Quebec	227 (30.5)	141 (30.4)	86 (30.6)	
	New Brunswick	16 (2.2)	12 (2.6)	4 (1.4)	
	Nova Scotia	17 (2.3)	12 (2.6)	5 (1.8)	
	Newfoundland	10 (1.3)	4 (0.9)	6 (2.1)	
	Prince Edward Island	4 (0.5)	2 (0.4)	2 (0.7)	
Language					.81
	English	613 (72.9)	374 (73.2)	239 (72.4)	
	French	228 (27.1)	137 (26.8)	91 (27.6)	
Work function impairment					<.001
	None	577 (70.4)	314 (62.9)	263 (81.9)	
	Mild	199 (24.3)	146 (29.3)	53 (16.5)	
	Moderate	41 (5)	36 (7.2)	5 (1.6)	
	Severe	3 (0.4)	3 (0.6)	0	

### Internet Usage

A majority of the participants reported use of Internet for personal reasons in the past 12 months, with the proportion (95.7%) among high-risk men being slightly higher than that among low-risk men (92.4%) (Table 2). The two groups did not differ in Internet use for searching medical information and in perceived usefulness of the Internet information in making decisions about health. However, high-risk men (83.4%) were more likely to report that it was important to access health resources on the Internet than low-risk men (75.0%).

**Table 2:** General and health-related Internet usage among men who were at different risk levels of major depression.

Internet use during the past 12 months		High risk, n (%)	Low risk, n (%)	*P*
Used Internet for personal use		489 (95.7)	305 (92.4)	.04
Hours of Internet each week (h)				
	<5	175 (35.8)	110 (36.1)	.81
	5-9	137 (28.0)	93 (30.5)	
	10-19	113 (23.1)	66 (21.6)	
	20-29	39 (8.0)	23 (7.5)	
	30-39	11 (2.3)	8 (2.6)	
	>40	14 (2.9)	5 (1.6)	
Used Internet on a mobile phone, tablet, or other mobile devices		408 (83.4)	235 (77.1)	.03
Used Internet for medical or health-related information		307 (62.7)	168 (55.1)	.08
How useful Internet helps you in making decisions about your health				
	Not useful	32 (10.7)	14 (8.5)	.90
	Unsure	41 (13.4)	28 (16.7)	
	Useful	231 (75.9)	125 (74.8)	
How important is it for you to be able to access health resources on the Internet				
	Not important	31 (10.1)	15 (8.9)	.01
	Unsure	20 (6.5)	27 (16.1)	
	Important	256 (83.4)	126 (75.0)	

### Preferred Design Features

Participants rated their level of interest in possible use of 17 different features that can be incorporated into the design of e-mental health programs. We ranked the preferred design features of e-mental health program in descending order (see Table 3). The top three features that were identified by high-risk men as things they would likely to use were: “information about improving sleep hygiene,” “practice and exercise to help reduce symptoms of stress and depression,” and “having access to quality information and resources about work stress issues.” The proportions of individuals endorsing the selected design features were significantly higher in the high-risk group than those in the low-risk group, except for “information about improving sleep hygiene” (Table 3).

**Table 3:** Proportions of preferred design features of e-mental health program in men who were at different risk levels of major depression.

Features		High risk, n (%)	Low risk, n (%)	*P*
**Likely use of …**				
	Information about improving sleep hygiene	313 (61.3)	181 (54.9)	.07
	Practice and exercise to reduce stress	303 (59.5)	127 (38.8)	<.001
	Quality information about work stress	295 (57.8)	146 (44.2)	<.001
	Setting personal goals and track them	277 (54.6)	128 (38.8)	<.001
	Watching videos online on how to deal with work and stress issues	272 (53.3)	125 (37.9)	<.001
	Being able to access a program via mobile phone or as an app.	264 (52.0)	127 (38.6)	<.001
	Direct referral to health professional to deal with work and stress issues in person	263 (51.7)	109 (33.0)	<.001
	Being able to ask questions and receive answers from mental health professional	253 (49.7)	120 (36.5)	<.001
	Self-help interactive program that provides info about work problems	244 (47.8)	96 (29.4)	<.001
	Being able to chart and track your mood	217 (42.6)	93 (28.5)	<.001
	A risk calculator predicting future risk of having major depression	215 (42.6)	94 (28.8)	<.001
	Access by phone to a trained coach to help with work stress	211 (41.3)	97 (29.6)	.001
	Information about anger management	211 (41.5)	88 (26.8)	<.001
	Receiving printed materials	167 (32.7)	78 (23.7)	.005
	Information delivered in game format	156 (30.7)	62 (18.8)	<.001
	Online peer connection	140 (27.5)	61 (18.5)	.003
	Online chat room	129 (25.3)	58 (17.6)	.009

### Preferred Design Features by Age and Language Among High-Risk Men

We estimated and compared the proportions of preferred design features by age groups and languages used in the interviews in men who were at high risk of MDE. The data showed that, compared to older participants, younger participants preferred access to a program through a smartphone or mobile app and that the information be delivered in game format (Table 4). Middle-aged men preferred receiving printed materials. The preferences for other design features did not vary by age.

English-speaking participants were more likely to use “practice & exercise to reduce stress,” “access a program via smartphone or an app,” and “being able to ask questions and receive answers from mental health professionals” than French-speaking men; French-speaking men were more likely to use “being able to chart and track your mood” than English-speaking participants (Table 5).

**Table 4:** Proportions of preferred design features of e-mental health program in men who were at high-risk levels of major depression by age groups.

Features		18-29 y, n (%)	30-49 y, n (%)	50-64 y, n (%)	65+ y, n (%)	*P*
**Likely use of …**						
	Being able to access a program via smartphone or as an app.	46 (63.0)	156 (55.3)	58 (41.7)	4 (28.6)	.003
	Receiving printed materials through mail	15 (20.6)	89 (31.3)	61 (43.6)	2 (14.3)	.002
	Information to be delivered in game format	36 (49.3)	84 (29.7)	33 (23.74)	3 (21.4)	.001

**Table 5:** Proportions of preferred design features of e-mental health program in men who were at high risk levels of major depression, by language used.

Features		English, n (%)	French, n (%)	*P*
**Likely use of …**				
	Practice and exercise to reduce stress	235 (63.2)	68 (49.6)	.006
	Being able to access a program via smartphone or as an app	206 (55.4)	58 (42.7)	.01
	Being able to ask questions and receive answers from mental health professionals	197 (52.8)	56 (41.2)	.02
	Being able to chart and track your mood	147 (39.5)	70 (51.1)	.02

A majority of participants considered our survey questions, about preferred design features, to be comprehensive and did not have other features to add. For the open-ended questions, some participants suggested that, in addition to the design features encompassed in the survey, other valuable features may be: easy to use (eg, “online information in a format that is simple to use.”), confidentiality (eg, “Privacy, somehow to ensure privacy”), credibility (eg, “having access to reliable information that's important to me”), and direct link to a professional (eg, “like some kind of call in line. Like a hotline … where you could access a live expert…. something personal”).

### Likely Use of E-Mental Health Programs

Among the participants, 69.0% reported “yes” or “maybe” to potentially using an e-mental health program to deal with work stress. The percentage was higher in the high-risk group (72.6%), those in the levels of higher education and personal income, younger age groups, and those working in mid and large companies, compared with their counterparts. No differences were found by language, marital status, and employment status (employee vs self-employed). High-MDE-risk participants who reported that they would not use an e-mental health program for dealing with stress were asked “what would make it difficult to use an e-health program?”. The reported barriers included perceived stigma associated with accessing e-mental health support (eg, “…social stigma, comfort of access,” “….workplace ignorance and what do they call that where you stereotype ...”), lack of personal interaction inherent to e-mental health (eg, “lack of personal face to face,” “… don’t see the value of it if you could talk to your family doctor…”), lack of time (eg, “…if it was time consuming…”), and lack of knowledge (eg, “Well the fact that I don’t know what an e health program is makes it difficult. I’m not sure that (laughs)”).

## Discussion

### Principal Results

One key finding of this study was that 62.7% participants who were at high risk of having MDE had used the Internet for health information in the 12 months prior to the survey. This percent is slightly higher than a similar estimate from the 2012 Canadian Internet Use survey in which 60.8% men reported use of Internet for medical or health-related information [19]. Furthermore, more than 75% of high-MDE-risk men in our sample considered health information on the Internet to be useful in helping them make health decisions and more than 72% would use an e-mental health program to deal with work related stress. Given that men often delay help-seeking for mental health problems because of perceived stigma and gender norms, our results suggest that the privacy inherent to e-mental health programs makes e-mental health programs a promising tool for improving men’s mental health.

Acceptability of a tool is vital to evaluation of its effectiveness and implementation. Therefore, to develop e-mental health programs for men, it is critical to understand their preferred design features. It is enlightening to observe, from our survey, that “information about improving sleep hygiene” was the top design feature preferred by men, irrespective of their risk status. Individuals who are at high risk of MDE may be occupied by unhelpful thinking and look for strategies to solve the issues they encounter. Thus, it is not surprising that the second top feature they endorsed was “practice and exercise to help reduce symptoms of stress and depression” which is consistent with the principles of CBT, for example, changing unhelpful thinking and behaviors and problem-solving focus. We anticipated that CBT practices and educational information (“having access to quality information and resources about work stress issues”) would be needed by the participants, and this was demonstrated in this study. This also is consistent with the fact that most of the existing e-mental health programs, such as MoodGYM [20], were developed based on the CBT approach [21]. We found that men who were at high risk of having an MDE were more likely to have endorsed the design features than men who were at low risk. No age differences were found in preferred design features. English-speaking participants were more likely to use CBT techniques and an app and French-speaking participants were more likely to use mood-monitoring tools. These results indicate that e-mental health programs incorporating these preferred features are likely to be used by men who are at high risk of an MDE across age and English- or French-speaking categories.

### Comparison With Prior Work

Understanding the barriers to the use of e-mental health programs is also important for the development, evaluation, and implementation of the programs. Some features preferred by the participants reflect the concerns they have about e-mental health programs and potential barriers to the use. Based on our quantitative and qualitative data, confidentiality and privacy are the prominent concerns for high-risk participants. They were concerned about the consequences if others know that they use the program to deal with stress related issues (eg, perceived stigma). Other barriers include extent to which the program is easy to use and navigate, credible (eg, information is provided by health professionals), relevant to one’s personal situation, and interactive (eg, being able to communicate with a professional). Additionally, lack of time and knowledge about e-health are potential barriers reported by the participants. Schneider et al [22] investigated users’ views of an online CBT program (MoodGYM) in a randomized controlled trial. Wetterlin et al’s [23] cross-sectional study examined youth expectations for mental health websites. Both studies reported preferences and perceived barriers that are consistent with the results of our survey.

### Limitations

Our study has several limitations. First, the survey data relied on self-report. Therefore, reporting and recall biases are possible. Second, our target population is Canadian working men who were aged 18 and older. As compared to men in the Canadian workforce in 2014, our sample was slightly older. The proportion of participants aged 18 to 29 in this study was 12.1%, whereas it was 20.2% in the Canadian workforce. Therefore, the proportions of some design features by age groups could have been overestimated or underestimated because of potential selection bias. Given the increasing use of mobile phones in young adults, future studies may investigate strategies for recruiting young adults through mobile phones. Finally, our survey collected self-reported qualitative information about barriers to the use of e-mental health programs. The qualitative information should be considered preliminary. More studies are needed to provide definitive answers.

### Conclusions

There is a pressing need for developing innovative strategies for prevention of depression in men. This is a challenging endeavor, given the gender norms and social stigma against depression and help seeking among men. E-mental health holds potential as it can be confidential, easily accessible, and economic if designed appropriately. More studies are needed to examine preferred design features and the barriers to use in different populations so that e-mental health strategies that meet the needs of different age groups and personal backgrounds can be developed.
